# Corrigendum

**DOI:** 10.1002/rcr2.486

**Published:** 2019-09-06

The authors would like to draw the reader's attention to an error in the following article:

Levin K, McClean C, Hoy R. (2019) Artificial stone‐associated silicosis: clinical‐pathological‐radiological correlates of disease. Respirology Case Reports, 7(7); e00470. https://doi.org/10.1002/rcr2.470

In the Discussion section, Reference 3 was inadvertently cited in the following sentence:

Pathology from the 13 sandblasters with silicoproteinosis was derived from bronchoalveolar lavage samples and autopsy specimens.

The authors apologize for this error and any confusion it may have caused.

